# From Neglected Desert Vine to Promising Bio-Resource: Phytochemistry, Biological Activities, Poultry Applications, and Ecological Significance of *Citrullus colocynthis* (L.) Schrad.

**DOI:** 10.3390/biology15171448

**Published:** 2026-08-24

**Authors:** Hanan Al-Khalaifah, Tahani Al-Surrayai, Sheikh Shreaz, Shikha Alzamel

**Affiliations:** Food Security Program, Environment and Life Sciences Research Center, Kuwait Institute for Scientific Research, P.O. Box 24885, Kuwait City 13109, Kuwait; tsuraia@kisr.edu.kw (T.A.-S.); szamel@kisr.edu.kw (S.A.)

**Keywords:** *Citrullus colocynthis*, phytochemistry, biological activities, sustainable agriculture, poultry nutrition, ecological restoration

## Abstract

*Citrullus colocynthis* (L.) Schrad., a drought-tolerant member of the Cucurbitaceae family, has been used in traditional medicine for centuries and has recently attracted increasing interest because of its potential pharmacological, agricultural, and ecological applications. This review examines current information on the plant’s distribution, ethnomedicinal uses, phytochemical composition, biological activities, applications in poultry, and ecological significance. Its bioactive constituents include cucurbitacins, flavonoids, phenolic compounds, alkaloids, glycosides, fatty acids, and tocopherols. These compounds have been associated with anticancer, antidiabetic, antimicrobial, antioxidant, anti-inflammatory, neuroprotective, hepatoprotective, and other biological activities, although most of the available evidence comes from in-vitro and preclinical studies. Initial findings also indicate that the plant may have value as a phytogenic feed additive in poultry and as a drought-tolerant species for soil stabilization, rangeland rehabilitation, and desertification control. However, the practical application of these findings is still constrained by differences in research methods, variation in phytochemical composition, limited clinical evidence, and the plant’s narrow therapeutic window and dose-dependent toxicity. Consequently, its therapeutic effectiveness and suitability for routine agricultural use cannot yet be established with certainty. Future research should focus on phytochemical standardization, quality control, thorough toxicological assessment, mechanistic studies, and well-designed clinical and animal trials using standardized preparations. Collectively, the available evidence indicates that *C. colocynthis* is a promising source of bioactive compounds with potential pharmaceutical, veterinary, and ecological applications, although rigorous scientific validation is required before its safe and effective utilization can be recommended.

## Simple Summary

*Citrullus colocynthis* (bitter apple) is a drought-tolerant medicinal plant traditionally used to treat various disorders and increasingly investigated for its pharmacological, agricultural, and ecological potential. Available studies suggest that its bioactive compounds possess diverse biological activities, while preliminary evidence indicates possible applications in poultry nutrition and rangeland restoration. Despite a rich history in traditional medicine, its modern application is severely restricted by inherent organ toxicity, extreme bitterness, invasive growth habits, and highly variable bioactive compound concentrations that complicate safe dosage standardization. To safely unlock the plant’s full therapeutic and agricultural value, future research must urgently prioritize toxicity reduction strategies, establish standardized extraction protocols, and conduct robust clinical and feeding trials.

## 1. Introduction

**Figure 1:**
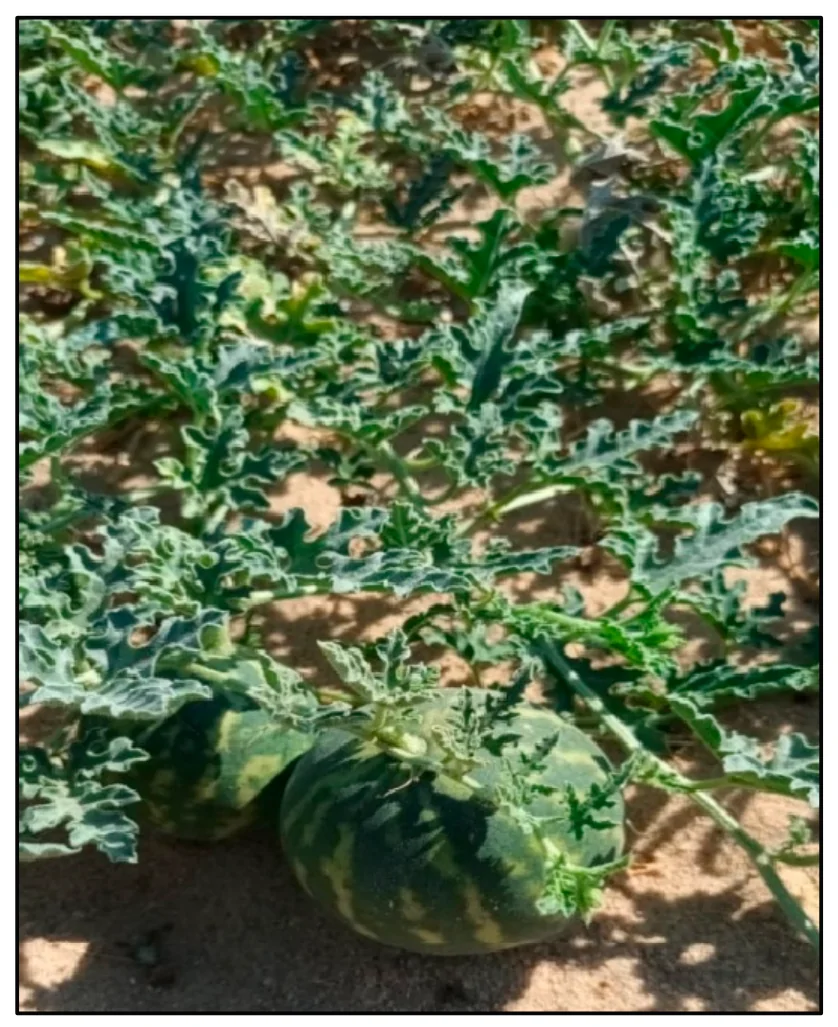
*C. colocynthis* growing in the Kuwait desert, showing the plant and fruit.

*Citrullus colocynthis* (L.) Schrad., the bitter apple, has received central attention due to its incredible scope of therapeutic uses, potential in sustainable rangeland development, and its unmatched success in dry and semi-arid soil habitats [1]. The plant is well-established in the sands of the desert of the Arabian Peninsula, including in the Gulf area [2], arid areas of Africa, Asia, and the Mediterranean, where it has acclimatized to both the climatic and soil extremes, indicating its possible use in ecological rehabilitation and in farming in climatically robust environments [1,3]. *C. colocynthis* has been found in the arid deserts of Kuwait (Figure 1), where the plant grows in sandy soils and sustains the harsh climatic conditions in the area [4]. The ancient medical uses of *C. colocynthis* are deeply rooted in the ancient forms of medicinal systems where the fruits, seeds, and leaves of this plant are used in the treatment of gastrointestinal disorders, diabetes, inflammation, and other diseases due to its rich phytochemical composition of alkaloids, flavonoids, cucurbitacins, and polyphenols [5,6].

**Figure 2:**
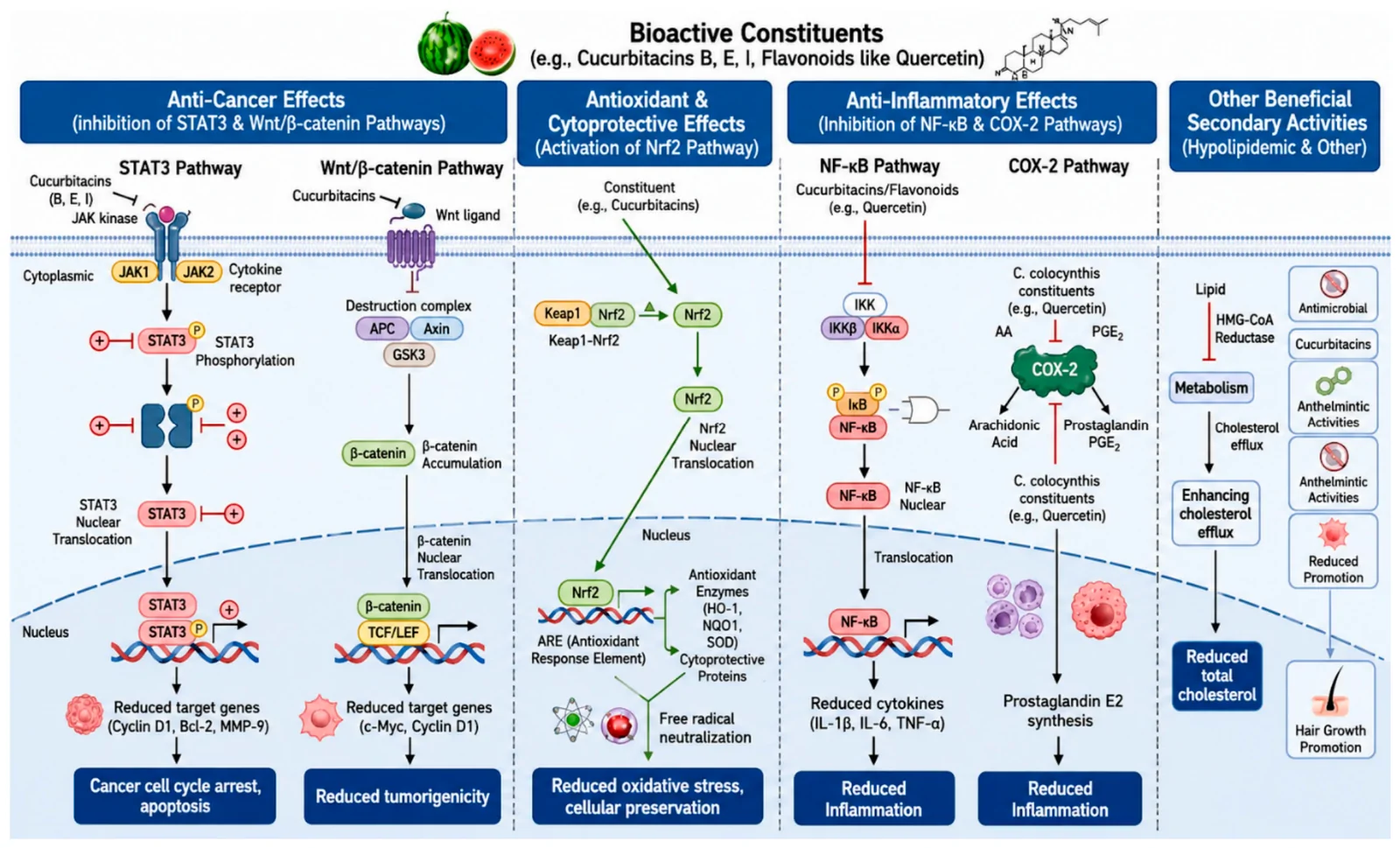
Mechanistic overview of the principal molecular targets and signaling pathways (including STAT3, Wnt/β-catenin, Nrf2, NF-κB, and COX-2) modulated by the major bioactive constituents of *C. colocynthis*, alongside a summary of secondary biological activities.

While several reviews have previously detailed the ethnopharmacology, phytochemistry, and pharmacological activities of *C. colocynthis* [1,7,8], this review pursues a different aim, rather than summarizing pharmacological effects as an isolated inventory, it integrates this chemical and pharmacological evidence with the plant’s comparatively underexamined applications in poultry nutrition and arid-land ecological restoration. A substantial gap still exists between our understanding of the plant’s phytochemical composition and its translation into safe, standardized applications. This gap is particularly important because unresolved concerns regarding toxicity continue to restrict its practical use. To illustrate these cellular mechanisms, Figure 2 presents a dedicated mechanistic diagram explicitly mapping the principal molecular targets and signaling cascades (including the STAT3, Wnt/β-catenin, Nrf2, NF-κB, and COX-2 pathways) modulated by the major bioactive constituents of *C. colocynthis*; these findings are critically reassessed in Section 5.

The urgent global challenge of efficiently utilizing resilient plant species in arid and semi-arid zones is underscored by the fact that nearly 41% of the earth’s surface comprises such ecosystems, which face severe threats like soil degradation, biodiversity loss, and advancing desertification [9,10]. *C. colocynthis* is an important native plant in Kuwait [11], uniquely adapted to the harsh environments, this plant can serve as sustainable resource for soil stabilization, forage, and ecological restoration in this region [12]. However, despite of its value, this plant is often overlooked in ecological, medicinal, and agricultural contexts [13]. It contributes significantly to the local biodiversity as a perennial herbaceous vine prevalent across sandy dunes and arid regions of the country [12,14,15]. This oversight brings into question how highly stress-tolerant plants can contribute to global goals in food security, climate resilience, and natural resource management. Native desert plants can serve as climate-adapted feed resources that support food security and reduce dependence on conventional inputs in arid regions [16]. Rising temperatures remain a major constraint on livestock productivity, reinforcing the value of heat-resilient arid-zone resources [17].

Currently, most remedial efforts in arid zones emphasize well-characterized crops and medicinal plants that require favorable soil and climatic conditions. Globally, staple drought-tolerant grains such as barley, sorghum, and millet are routinely promoted to counteract soil degradation and food insecurity [18,19], but research and practical focus seldom extend to non-traditional, extreme-resilience species like *C. colocynthis*. Similarly, important native plants of the Arabian Peninsula such as *Haloxylon salicornicum* (Rimth), *Lycium shawii* (Awsaj) and *Salsola imbricata* (Saltwort, Milayah) have well established pharmaceutical profiles and dominate integrative medicine [20,21,22], whereas the potential of *C. colocynthis* to match or surpass their benefits in resource-limited, arid settings remain inadequately explored. The outstanding feature of *C. colocynthis* is its phenomenal tolerance to drought, the ability to grow in marginal soils and diverse phytochemistry [23]. Its well-known folk medicine applications such as treatment of diabetes, gastrointestinal disturbances, and inflammation are due to its strong bioactive compounds whereas its antioxidant, anti-inflammatory, antimicrobial and anticancer properties are justified in recent studies [1,5,24]. Also, for poultry production systems, native plants from Kuwait have already been explored as important poultry feed resources [16]. *C. colocynthis* when used in poultry has been reported to have some promising outcomes as a natural feed supplement, and improves the performance of broilers, their immune functions and antioxidant properties in a stressed environment [25,26]. These traits of robustness and adaptability make this plant a potential option in the restoration of rangelands, livestock fodder, pest control, and even soil conservation programs in the degraded landscapes [27]. Nevertheless, challenges exist, the most prominent being the liver and kidney toxicity demonstrated [28,29], absence of standard procedures in the safe usage and the undesirable bitterness and invasive growth limiting its use in fodder and agricultural systems [1,30]. The risk of an undesirable impact, especially on livestock and unintentional ingestion by humans, is the reason why stringent safety measures and control measures are required.

This review presents an integrated and critical assessment of the medicinal, agricultural, and ecological importance of *C. colocynthis*, while emphasizing the connections among these areas of research. In contrast to earlier reviews that have generally examined specific topics, such as phyto-chemistry or pharmacology, the present work follows a structured analytical approach. It begins by exploring the ecological and molecular mechanisms underlying the plant’s remarkable drought tolerance, including recent genomic developments such as the chromosome-level genome assembly [23]. The review then examines its phytochemical profile in relation to the reported pharmacological effects and associated toxicological risks. Finally, it considers the potential use of *C. colocynthis* in sustainable poultry nutrition and the restoration of degraded ecosystems. By bringing these perspectives together, this review offers a balanced and current evaluation of *C. colocynthis* as a multifunctional, climate-resilient bio-resource, while also stressing the need to carefully address its toxicological limitations.

## 2. Botanical Characteristics, Geographical Distribution, and Its Adaptation to Arid Environments

*C. colocynthis* is a perennial, herbaceous, trailing vine belonging to the Cucurbitaceae family. It is widely distributed throughout the arid and semi-arid regions of North Africa, the Arabian Peninsula, and South Asia, where it commonly grows in sandy soils, rocky areas, and wastelands [31,32,33,34,35]. Archaeological records suggest that the species was known in Egypt and the Near East as early as 4000 BC and later spread across the Mediterranean region through historical trade routes [36].

The plant has angular, coarsely hairy stems that may extend several metres. Its leaves are alternate, deeply lobed, and generally measure 5–10 cm in length. Solitary, yellow, unisexual flowers develop in the axils of the leaves [37]. The fruit is a hard, spherical, indehiscent berry measuring approximately 7–10 cm in diameter [7]. It is typically green with yellow markings, turning yellow as it matures and dries. The seeds are brown, smooth, ovoid, and compressed, with an average length of about 6 mm. Each fruit contains approximately 200–300 seeds, which account for nearly 75% of the total fruit mass [8].

### 2.1. Morphological Adaptations

*C. colocynthis* can withstand prolonged periods of water shortage partly because of its deep, perennial taproot, which allows it to access water from deeper soil layers. In addition, the thick, hydrophobic cuticle and dense trichomes present on its leaves and stems help reduce water loss through transpiration, thereby contributing to its drought tolerance [7,38,39].

### 2.2. Physiological Mechanisms

The species also shows considerable tolerance to heat and salinity. During heat and drought stress, it accumulates proline, which functions as an osmoprotectant, and strengthens its antioxidant defense systems. These responses help limit oxidative damage and maintain cellular homeostasis [40]. Its extensive root system may further support soil stabilization and help reduce wind erosion in desert environments [41].

### 2.3. Molecular and Genomic Adaptations

Recent genomic research has provided further insight into the molecular mechanisms associated with drought adaptation in *C. colocynthis*. The International Center for Biosaline Agriculture (ICBA) developed a chromosome-level genome assembly containing more than 23,327 annotated genes, thereby providing a valuable resource for studying xerophytic adaptations [23]. Transcriptomic analyses identified 5038 cDNA sequences, including 2545 genes that were significantly upregulated under drought conditions. These genes were mainly associated with transcriptional regulation, signal transduction, detoxification, citrulline metabolism, and phytohormone signaling pathways involving abscisic acid, salicylic acid, and jasmonic acid [41,42].

Although these findings have advanced current understanding of drought adaptation in the species, additional functional studies are needed to confirm the roles of these genes and determine whether this knowledge can be applied to the development of drought-resilient crops.

## 3. Traditional Use

**Figure 3:**
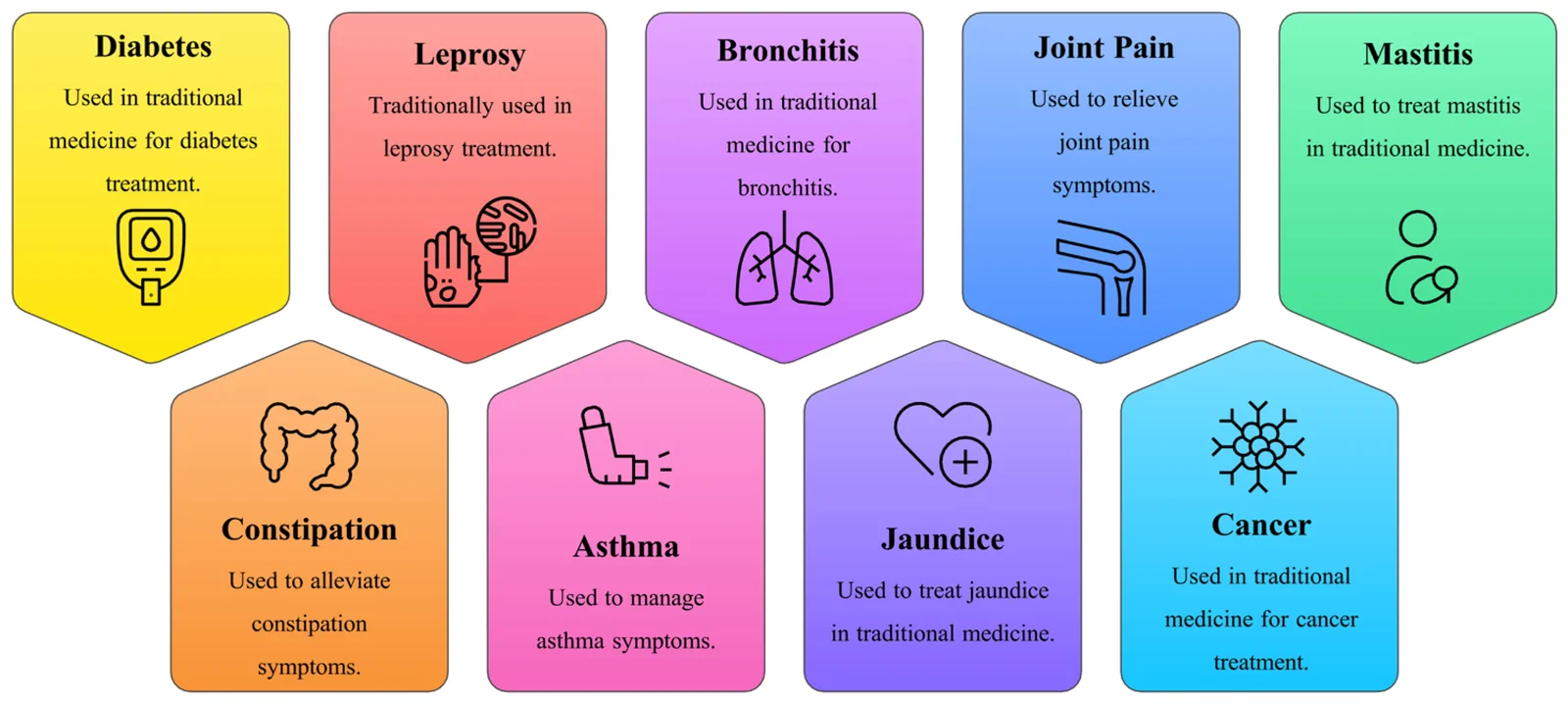
Traditional uses of *C. colocynthis*.

The medicinal use of *C. colocynthis* has long been documented in the traditional healing systems of North Africa, the Middle East, and South Asia [7,43,44,45]. Although the plant has been widely used in ethnomedicine, much of the evidence for its therapeutic efficacy derives from traditional knowledge and ethnobotanical observations rather than well-controlled clinical studies [7,46] (Figure 3). Accordingly, traditional claims should be interpreted with appropriate caution.

When categorized according to preparation methods and geographical regions, the most consistently reported traditional application of *C. colocynthis* is as a potent gastrointestinal purgative [7,47]. In Saudi Arabia and Jordan, fruit preparations are used to relieve stomach pain, while fresh seeds (locally known as *Handal*) have traditionally been consumed to manage severe diarrhea, colic, and gastroenteritis [47,48,49,50]. In Morocco and Tunisia, fruit decoctions are commonly used in traditional medicine for managing metabolic and cardiovascular disorders, including diabetes and hypertension [51,52,53,54]. Similarly, topical application of seed oil has been reported in East Africa and Mediterranean communities for treating localized inflammation, rheumatic pain, and dermatological disorders [55,56,57]. Several of these traditional applications, particularly the antidiabetic and topical anti-inflammatory uses, have subsequently been investigated in experimental in-vitro and in-vivo studies. However, these findings should be regarded as preclinical evidence and should not be interpreted as confirmation of clinical efficacy [58,59,60].

In contrast, traditional claims regarding the treatment of severe systemic diseases, including cancer, leprosy, jaundice, asthma, and mastitis, remain largely anecdotal and are supported by limited experimental evidence [61,62]. Consequently, these claims should be considered preliminary until validated through well-designed pharmacological and clinical investigations.

Traditional use must also be interpreted in the context of the plant’s well-documented toxicological profile. *C. colocynthis* possesses a narrow therapeutic index, and traditional reports describe acute intoxication following the consumption of raw or excessive amounts of the fruit, particularly immature fruits, resulting in severe vomiting, hematochezia, and, in some cases, fatal outcomes [47,55,63]. To minimize these adverse effects, several traditional preparation methods involve detoxification or dilution practices. For example, in southern Punjab, Pakistan, dried fruit powder is mixed with jaggery to prepare deworming boluses rather than being administered alone [59]. Overall, although *C. colocynthis* remains an important ethnomedicinal species, its documented toxicity highlights the need for standardized preparations, dose optimization, and rigorous safety evaluation before its broader therapeutic application.

## 4. Phytochemical Analysis

The phytochemical composition of *C. colocynthis* is chemically diverse, with distinct classes of secondary metabolites predominantly distributed among specific plant parts, including the fruits, leaves, seeds, and roots. Recent studies have progressed beyond preliminary phytochemical screening by employing advanced analytical techniques, such as high-performance liquid chromatography (HPLC), nuclear magnetic resonance (NMR), and mass spectrometry (MS), enabling the reliable isolation and structural characterization of numerous bioactive compounds (Table 1) [44,64,65,66,67,68,69,70,71,72,73,74,75].

### 4.1. Cucurbitacins and Glycosides

Cucurbitacins constitute the major group of bioactive metabolites identified in *C. colocynthis*. These tetracyclic triterpenes, including cucurbitacins B, E, I, J, K, and L, are found mainly in the fruits and leaves. Advanced spectroscopic techniques, particularly NMR and MS, have confirmed the structures of several glycosylated derivatives, including colocynthosides A and B and cucurbitacin 1,2-*O*-β-D-glucopyranoside [64,65,66,67,68,69,70]. Structural differences, especially in oxygenation patterns and glycosylation, are considered important factors influencing their biological activities [76].

### 4.2. Flavonoids and Phenolic Acids

Phenolic acids and flavonoids are widely distributed in the roots, leaves, and fruit pulp of *C. colocynthis*. The major identified compounds include gallic acid, chlorogenic acid, ferulic acid, vanillic acid, quercetin, myricetin, and catechin [44,73]. In addition, flavonoid glycosides, such as isosaponarin and isovitexin, have been isolated from the fruits and structurally characterized using HPLC [72].

### 4.3. Alkaloids, Fatty Acids, and Steroids

The seeds constitute an important source of fixed oils enriched with essential fatty acids, including linoleic, oleic, palmitic, and stearic acids, together with tocopherols, which have been characterized using NMR and Fourier-transform infrared spectroscopy (FTIR) [75]. Quinoline-type alkaloids have been consistently identified in the fruits, whereas phytosterols such as spinasterol have been isolated from the leaves [71,74]. Collectively, these metabolites reflect the considerable chemical diversity of *C. colocynthis* and provide a biochemical foundation for many of its reported biological activities.

**Figure 4:**
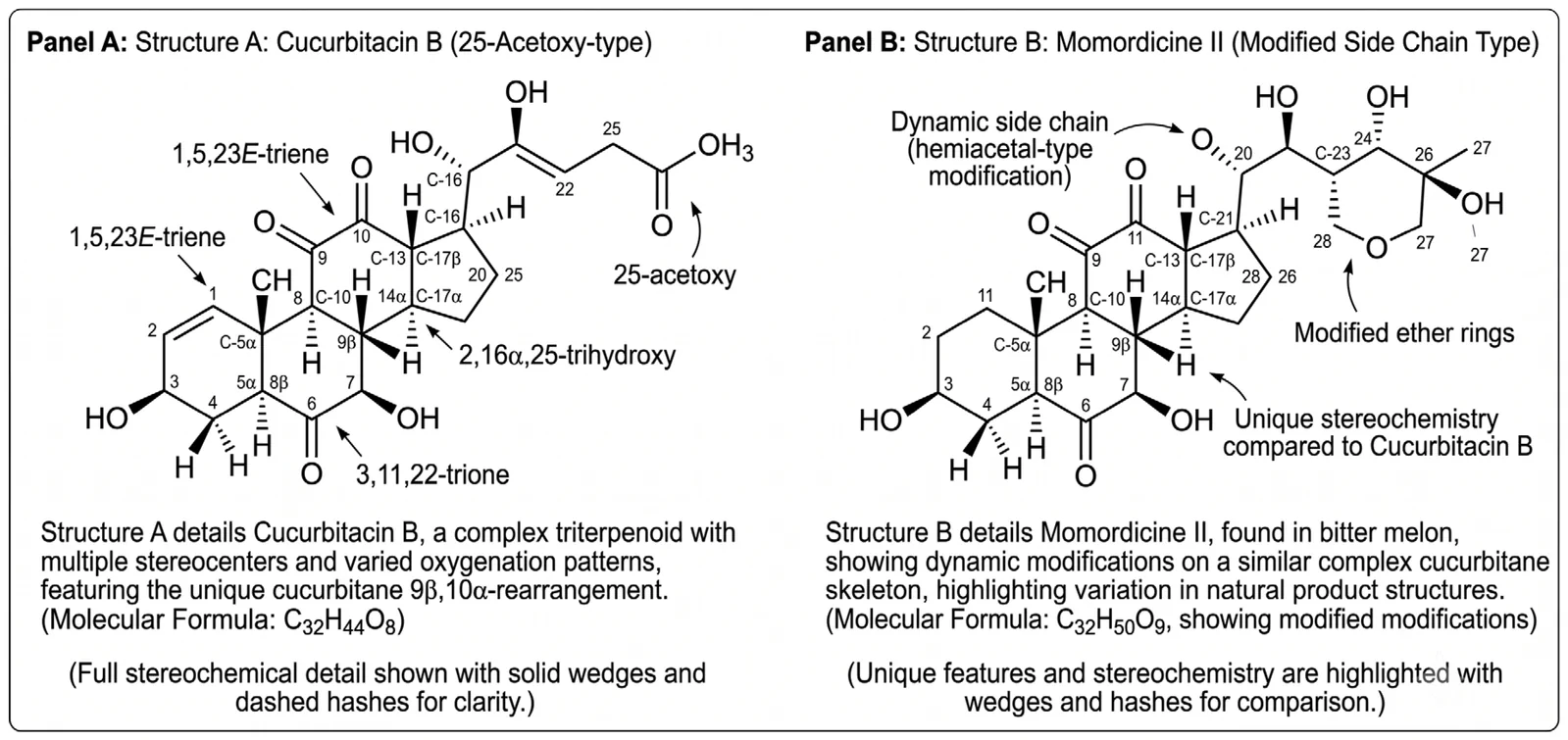
Chemical structures of the major bioactive phytochemicals of *C. colocynthis*, featuring standardized formatting and explicit stereochemical representations.

A detailed examination of the chemical characteristics and structure–activity relationships of its predominant metabolites is essential for gaining a comprehensive understanding of the plant’s bioactivity profile (Figure 4). The pharmacological and toxicological properties of *C. colocynthis* are largely attributed to cucurbitacins, which are highly oxygenated tetracyclic triterpene structures and their glycosylation patterns are believed to influence their interactions with biological targets [64,65,66,67,68,69]. These structural characteristics may contribute to the diverse biological activities reported in experimental studies, including cytotoxic and biocidal effects, and also account for their recognised toxicological potential [64,70]. In contrast, the antioxidant activity of *C. colocynthis* has been primarily associated with phenolic acids and flavonoids, including quercetin and myricetin [72,73]. The hydroxyl groups present on their aromatic rings facilitate electron and hydrogen atom donation, thereby contributing to free radical scavenging activity. Additionally, the seed oil is rich in polyunsaturated fatty acids, particularly linoleic and oleic acids, which may contribute to the hypolipidemic effects reported in experimental studies and support the seeds’ nutritional value for potential poultry feed applications [75,77]. Overall, the diverse biological properties of *C. colocynthis* are likely the result of the combined actions of multiple phytochemical classes rather than a single group of compounds.

## 5. Biological Activity

### 5.1. Anti-Cancer Activity

Extracts and isolated phytochemicals of *C. colocynthis* have demonstrated in-vitro cytotoxic activity against a wide range of human cancer cell lines, including breast, cervical, colon, liver, leukemia, brain, prostate, kidney, lung, and melanoma cells (Table 2) [64,67,70,78,79,80,81,82,83]. However, these findings should not be interpreted as evidence of clinical anticancer efficacy. Furthermore, several early studies did not report quantitative data for standard reference drugs, limiting direct comparisons of the potency of *C. colocynthis* extracts and isolated compounds (Table 2). In addition, the concentrations employed varied considerably among studies, with some crude extracts showing activity only at relatively high concentrations (mg/mL), whereas purified cucurbitacins were active at lower concentrations (μg/mL or μM). These differences should be considered when interpreting the reported cytotoxic effects.

Among the identified phytochemicals, cucurbitacin B has demonstrated dose-dependent cytotoxic activity against Hep-2 human laryngeal carcinoma cells by inducing G2-phase cell-cycle arrest, promoting apoptosis, and downregulating the expression of *p*-STAT3, cyclin B1, and Bcl-2 [84]. It has also exhibited in-vitro inhibitory activity against glioblastoma multiforme cells [85]. Other cucurbitacins, including cucurbitacins E, I, and L, have likewise shown cytotoxic activity in different cancer cell models [64,70]. In addition, methanolic extracts of *C. colocynthis* have been reported to modulate cyclin-dependent kinase (cyclin–CDK) signaling pathways [78], whereas ethanol and acetone fruit pulp extracts reduced the expression of anti-apoptotic genes (*BCL2* and *BCLXL*) and increased the expression of pro-apoptotic genes (*BAX* and *caspase-3*) in metastatic MDA-MB-231 breast cancer cells [79]. Extracts have also been reported to inhibit Wnt/β-catenin signaling, suggesting potential effects on pathways associated with tumor progression [86].

Despite these promising mechanistic findings, the available evidence is still based largely on in vitro studies. Most investigations have not thoroughly assessed the therapeutic selectivity of *C. colocynthis* extracts or compounds toward cancer cells compared with normal cells, and it remains unclear whether the effective concentrations reported in vitro can be achieved safely in vivo. Therefore, the current evidence is insufficient to support the development of *C. colocynthis* as an anticancer therapeutic agent. Well-designed in vivo studies, followed by pharmacokinetic and toxicological evaluations and, ultimately, clinical investigations, are necessary to establish its efficacy, safety, and therapeutic potential.

### 5.2. Broad-Spectrum Biocidal and Antiparasitic Activity

Extracts and isolated phytochemicals from *C. colocynthis* have been evaluated against a wide range of arthropods, molluscs, and protozoan parasites, including agricultural pests, disease vectors, mites, and intermediate-host snails (Table 3) [71,87,88,89,90,91,92,93,94]. These studies demonstrate diverse biocidal activities; however, comparisons among studies remain difficult because different extraction methods, experimental protocols, and biological endpoints were employed. Moreover, several studies did not include standard reference agents, limiting the assessment of the relative potency of *C. colocynthis* extracts and isolated compounds.

Leaf and fruit extracts have demonstrated insecticidal and larvicidal activity against medically important mosquito vectors, including *Aedes aegypti*, *Culex quinquefasciatus*, and *Anopheles stephensi*. Fatty acids such as linoleic and oleic acids have also been associated with ovicidal and larvicidal effects in these mosquito species [89,90,91,93]. In addition, *C. colocynthis* extracts have shown moderate acaricidal activity against *Tetranychus urticae* and molluscicidal activity against the intermediate host snail *Galba truncatula*, effects that have been primarily attributed to cucurbitacin derivatives [71,92]. Methanolic fruit extracts have also demonstrated in-vitro antiprotozoal activity against chloroquine-resistant *Plasmodium* spp. and *Toxoplasma gondii* [87,88].

Overall, the available evidence indicates that *C. colocynthis* possesses insecticidal, acaricidal, molluscicidal, and antiprotozoal activities across multiple experimental systems. However, the reported activities are predominantly derived from laboratory-based studies, and differences in experimental design limit direct comparisons among investigations. Consequently, additional studies employing standardized bioassays, appropriate positive controls, and field validation are required before these findings can be translated into practical applications for integrated pest and vector management.

### 5.3. Anti-Microbial Activity

Various extracts and phytochemicals of *C. colocynthis* have been evaluated for in-vitro antimicrobial activity against a range of bacterial and fungal pathogens (Table 4) [95,96,97,98,99,100,101,102]. However, comparison of the reported activities is challenging because the experimental methodologies vary considerably among studies. Moreover, several investigations did not include standard antibiotic controls, limiting the assessment of the relative potency of *C. colocynthis* extracts and isolated compounds.

Most available studies employed qualitative screening methods, such as agar well diffusion and disk diffusion assays, which measure the zone of inhibition (ZOI) [96,98,100]. Although these assays provide preliminary evidence of antimicrobial activity, ZOI values are strongly influenced by the diffusion characteristics of test compounds in agar and therefore cannot be directly correlated with antimicrobial potency or clinical efficacy. In contrast, a smaller number of studies determined MIC and MBC values, and mechanistic actions which provide more reliable quantitative measures of antimicrobial activity [95,97,99].

Current evidence indicates that the antibacterial activity of *C. colocynthis* has been investigated more extensively than its antifungal activity. In-vitro inhibitory effects have been reported against both Gram-positive bacteria, including *Staphylococcus aureus* and *Bacillus subtilis*, and Gram-negative bacteria, such as *Escherichia coli* and *Klebsiella pneumoniae* [96,97,98]. By comparison, evidence for antifungal activity against organisms such as *Candida albicans* and *Aspergillus fumigatus* is limited and is based primarily on qualitative ZOI assays. Consequently, these findings require confirmation through standardized MIC/MFC assays and additional mechanistic studies [96,100].

Although the available data demonstrate in-vitro antimicrobial activity, their clinical significance remains uncertain. Most studies have not evaluated activity against well-characterized multidrug-resistant clinical isolates or systematically compared the results with standard antimicrobial agents. Furthermore, standardized susceptibility testing, time-kill kinetics, mechanism-of-action studies, toxicity assessments, and in-vivo efficacy evaluations remain limited. Therefore, while *C. colocynthis* represents a promising source of antimicrobial phytochemicals, additional well-designed studies are required before its therapeutic potential against infectious diseases, particularly those caused by multidrug-resistant pathogens, can be reliably established.

### 5.4. Anti-Oxidant Activity

Various extracts and isolated phytochemicals of *C. colocynthis* have demonstrated antioxidant activity in several in-vitro biochemical assays [103,104]. However, interpretation of these findings requires careful distinction between crude extracts and purified metabolites, as well as consideration of the different antioxidant assays employed. The reported assays evaluate different aspects of redox activity, including free-radical scavenging using 2,2-diphenyl-1-picrylhydrazyl (DPPH) and 2,2′-azino-bis(3-ethylbenzothiazoline-6-sulfonic acid) (ABTS) assays, nitric oxide (NO) scavenging, and inhibition of linoleic acid peroxidation, each reflecting distinct antioxidant mechanisms rather than overall antioxidant capacity.

Methanolic extracts of *C. colocynthis* exhibited 89.3% inhibition at 50 μg/mL in the DPPH assay and 86.0% inhibition in the NO scavenging assay [103]. Similarly, ethanolic fruit extracts inhibited linoleic acid peroxidation by 76.5% [73]. However, these values should be interpreted in comparison with the activities of standard antioxidants, such as ascorbic acid, Trolox, or butylated hydroxytoluene (BHT), when evaluated under comparable experimental conditions. In addition, differences in extraction procedures, assay protocols, and expression of results limit direct comparisons among studies.

The antioxidant activity of crude methanolic and ethanolic extracts is generally attributed to their phenolic acids, flavonoids, and other redox-active phytochemicals [73]. In contrast, purified cucurbitacin derivatives have demonstrated measurable ABTS radical-scavenging activity, with an IC_50_ value of 145.0 μM [104], suggesting that these compounds may also contribute to the antioxidant properties of the crude extracts. Nevertheless, the relative contribution of individual metabolites remains insufficiently characterized.

Overall, the available evidence identifies *C. colocynthis* as a potential source of antioxidant phytochemicals. However, the reported activities are derived predominantly from in-vitro chemical assays, which cannot fully predict biological efficacy under physiological conditions. Therefore, further in-vivo studies are required to establish the bioavailability, pharmacokinetic behaviour, and therapeutic relevance of these antioxidant effects.

### 5.5. Anti-Inflammatory Activity

The gastrointestinal adverse effects associated with prolonged use of non-steroidal anti-inflammatory drugs (NSAIDs) have encouraged the search for plant-derived alternatives with improved safety profiles [105]. Although *C. colocynthis* has traditionally been used for inflammatory conditions, experimental evidence supporting its anti-inflammatory activity remains limited. An aqueous fruit extract significantly reduced carrageenan-induced paw edema in rats at a dose of 4 mg/kg, providing preliminary evidence of anti-inflammatory activity in this experimental model [54].

Despite this observation, the available evidence is insufficient to establish the efficacy of *C. colocynthis* as a broadly effective anti-inflammatory agent. The current data are limited to a small number of preclinical studies, and the underlying molecular mechanisms, optimal dosing, reproducibility, and long-term safety remain poorly characterized. Furthermore, the documented toxicological profile of *C. colocynthis* warrants careful consideration when interpreting these findings. Consequently, the available evidence does not support the use of *C. colocynthis* as an alternative to conventional NSAIDs. Additional well-designed in-vivo studies, together with comprehensive dose–response, pharmacokinetic, and toxicological evaluations, are required before its therapeutic potential for inflammatory disorders can be reliably established.

### 5.6. Hypolipidemic Activity

Hypolipidemic effects of *C. colocynthis* have been reported in both experimental animal models and limited human studies; however, the available evidence should be interpreted cautiously because of substantial differences in the plant preparations, dosages, and study designs employed. In a hyperlipidemic rabbit model, administration of an ethanolic extract (1.2 g/kg/day) restored serum cholesterol levels to near-normal values [77]. In contrast, a clinical study involving hyperlipidemic non-diabetic subjects reported significant reductions in serum triglyceride and cholesterol concentrations following daily administration of 300 mg of raw seed powder [106]. Because ethanolic extracts and raw seed powder differ considerably in their phytochemical composition, bioavailability, and standardization, these findings should not be considered directly comparable or interpreted as a single body of evidence.

The mechanisms underlying the reported hypolipidemic effects remain poorly understood. It has been proposed that *C. colocynthis* may influence lipid metabolism through reduced intestinal lipid absorption, modulation of hepatic cholesterol synthesis, enhanced lipoprotein catabolism, or the antioxidant activity of its phenolic and other redox-active metabolites. However, these mechanisms remain largely hypothetical and have not been conclusively demonstrated in experimental studies.

Despite these preliminary findings, the clinical relevance of *C. colocynthis* for the management of hyperlipidemia remains uncertain. The available human evidence is limited, and the plant’s well-documented narrow therapeutic index and potential gastrointestinal toxicity raise important safety concerns. Consequently, current evidence is insufficient to recommend *C. colocynthis* as a hypolipidemic therapeutic agent. Additional well-designed preclinical and clinical studies using standardized preparations are required to establish its efficacy, safety, optimal dosage, and long-term tolerability.

### 5.7. Anti-Allergic, Antihistamine, and Bronchoprotective Activity

Preliminary experimental studies have demonstrated that *C. colocynthis* possesses anti-allergic, antihistaminic, and bronchoprotective activities; however, these findings should be interpreted cautiously because they are based on a limited number of preclinical models [66,107]. Anti-allergic activity was evaluated using an in-vivo passive cutaneous anaphylaxis (PCA) mouse model, in which a methanolic extract (200 mg/kg) produced 72.5% inhibition of the PCA response, whereas the isolated compound cucurbitacin E-2-*O*-β-D-glucopyranoside produced 49.7% inhibition at the same dose [66]. These findings suggest inhibition of local immediate hypersensitivity reactions; however, the PCA model does not fully represent systemic allergic responses or clinical allergic disease.

The antihistaminic and broncho-protective activities of *C. colocynthis* have been investigated using isolated goat tracheal chain preparations and histamine-induced bronchoconstriction models in guinea pigs [107]. In these experimental systems, fruit extracts reduced tracheal smooth muscle contraction and prolonged the pre-convulsive dyspnea period in a dose-dependent manner, indicating potential broncho-dilatory and antihistaminic effects. Nevertheless, these experimental models provide only preliminary pharmacological evidence and cannot reliably predict clinical efficacy in complex respiratory disorders such as asthma.

Interpretation of these biological activities should also consider the documented toxicological profile of *C. colocynthis*. The methanolic extract was evaluated at a relatively high dose (200 mg/kg), and several cucurbitacin derivatives are known to possess a narrow therapeutic window. Consequently, the therapeutic potential of *C. colocynthis* should be balanced against its potential toxicity. Further studies are required to establish dose–response relationships, mechanisms of action, pharmacokinetic properties, and comprehensive safety profiles before clinical application can be considered [66,107].

### 5.8. Anti-Diabetic Activity

*C. colocynthis* has been investigated in in-vitro, in-vivo, and limited clinical studies for its potential metabolic-modulating effects (Table 5) [55,108,109,110,111,112,113,114,115,116,117,118]. However, the term “anti-diabetic activity” encompasses multiple pharmacological mechanisms, including antihyperglycemic, insulinotropic, anti-glycation, enzyme-inhibitory, and hypolipidemic effects. Therefore, the available evidence should be interpreted according to the level of experimental validation and the specific biological mechanisms involved.

In-vitro studies have provided mechanistic insights into the metabolic effects of *C. colocynthis*. Aqueous and hydroethanolic extracts of the seeds and fruit pulp stimulated insulin secretion from isolated rat pancreatic islets, indicating insulinotropic activity [110,112]. Additional studies have demonstrated inhibition of advanced glycation end-product formation, reflected by reduced HbA1c formation [109], as well as α-glucosidase inhibitory activity, suggesting a potential role in improving postprandial glycemic control [111].

Evidence from in-vivo studies is more variable because different plant parts, extraction methods, and phytochemical fractions have been evaluated. Glycosidic and saponin-rich fruit fractions demonstrated greater antihyperglycemic activity than crude alkaloidal fractions in experimental diabetic models [55]. Likewise, aqueous extracts of fruits and seeds have been reported to reduce blood glucose and triglyceride concentrations and alleviate selected diabetic complications [113,114]. However, considerable methodological heterogeneity, including differences in extraction methods, dosages, and experimental models that makes direct comparisons between studies difficult. This variation also emphasizes the need for standardized preparations and for identifying the bioactive constituents responsible for the observed effects.

Clinical evidence remains limited. Short-term studies in patients with type 2 diabetes have reported reductions in fasting blood glucose (FBG) and HbA1c following the administration of encapsulated *C. colocynthis* fruit powder or decoctions [115,116,117,118]. Nevertheless, these findings should be interpreted cautiously, as the available trials generally involved small sample sizes, brief intervention periods of 10–60 days, and limited safety assessments. In addition, the documented gastrointestinal toxicity and narrow therapeutic index of *C. colocynthis* raise important concerns about its long-term therapeutic use. Similar clinical studies have reported short-term improvements in glycemic parameters, but they do not establish long-term safety or efficacy.

Overall, the available evidence suggests that *C. colocynthis* contains bioactive constituents with potential antihyperglycemic and metabolic-modulating properties. Nevertheless, the current evidence remains methodologically heterogeneous, relies on diverse plant preparations, and lacks comprehensive long-term safety and efficacy data. Further well-designed preclinical and clinical studies using standardized preparations are required before its therapeutic potential in the management of type 2 diabetes can be established.

### 5.9. Neuropharmacological and Neuroprotective Activity

*C. colocynthis* has been investigated in several experimental models for its neuropharmacological and neuroprotective effects; however, these activities represent distinct pharmacological endpoints and should be interpreted separately [119,120,121,122,123,124]. The available evidence is derived primarily from preclinical studies, and its clinical relevance remains uncertain.

Fruit extracts of *C. colocynthis* have demonstrated anticonvulsant activity in pentylenetetrazol-induced seizure models in mice. Oral doses of 25 and 50 mg/kg delayed seizure onset and reduced seizure duration. These findings indicate anticonvulsant activity rather than direct neuroprotection, as suppression of seizure activity does not necessarily imply preservation of neuronal structure or function. Although these effects have been attributed to possible modulation of GABAergic and opioid signaling pathways, these mechanisms remain speculative because receptor-specific pharmacological studies have not yet been performed [120].

The plant has also been evaluated in experimental models of drug-induced extrapyramidal dysfunction. In haloperidol-treated rats, *C. colocynthis* extracts reduced oxidative stress, partially restored dopamine levels, and attenuated catalepsy and vacuous chewing movements [121,122,123,124]. These findings suggest symptomatic improvement in a pharmacologically induced extrapyramidal model rather than disease-modifying or antiparkinsonian activity in Parkinson’s disease.

Evidence for neuroprotection has been reported mainly in models of hyperglycemia-induced neuronal injury. Methanolic and ethanolic fruit extracts reduced oxidative stress and improved cell viability in PC-12 cells exposed to high-glucose conditions [122]. Similarly, protective effects against hyperglycemia-associated neuronal damage and cognitive impairment have been observed in streptozotocin-induced diabetic rats [123]. However, these findings are specific to metabolic models of neuronal injury and should not be extrapolated to progressive neurodegenerative disorders.

Overall, the available evidence suggests that *C. colocynthis* possesses anticonvulsant, neuromodulatory, and neuroprotective properties in selected experimental models. Nevertheless, the current evidence remains limited to preclinical studies, and the underlying mechanisms, therapeutic selectivity, long-term safety, and clinical efficacy remain insufficiently characterized. Further mechanistic investigations, standardized in-vivo studies, and clinical evaluations are required before its therapeutic potential in neurological disorders can be established.

### 5.10. Hair Growth Promotion

Hair loss encompasses a group of dermatological disorders with diverse etiologies, including androgenetic alopecia, alopecia areata, and chemotherapy-induced alopecia [125,126,127,128,129]. *C. colocynthis* has traditionally been used in ethnomedicine as a topical remedy for hair loss [130,131]; however, scientific evidence supporting this application remains limited and is based primarily on a small number of preclinical studies.

Two in-vivo studies have evaluated the hair growth-promoting potential of *C. colocynthis*. Ethanolic and petroleum ether extracts stimulated hair regrowth in denuded skin of albino mice when compared with 2% minoxidil [132]. In another study, a petroleum ether fruit extract increased hair follicle density and promoted the anagen phase in a testosterone-induced alopecia model, showing effects comparable to those of 2% and 5% finasteride under the experimental conditions employed [131].

Despite these encouraging findings, their clinical significance remains uncertain. The available evidence is based on only a limited number of animal studies, and important physiological differences between murine and human skin, including variations in hair follicle cycling and transdermal absorption that make it difficult to extrapolate these findings directly to clinical practice. Moreover, the extracts used in these studies were not chemically standardized, which limits reproducibility and makes comparisons with approved pharmacological agents challenging. Topical safety also remains inadequately investigated. Given the known toxicity of *C. colocynthis*, the possibility of dermal absorption and the occurrence of local or systemic adverse effects require careful evaluation. Therefore, well-standardized formulations, comprehensive toxicological studies, and controlled clinical trials are necessary before *C. colocynthis* can be considered a potential treatment option for hair loss.

### 5.11. Hepatoprotective Activity

The hepatoprotective potential of *C. colocynthis* remains unclear, as the plant is also known for its dose-dependent hepatotoxic effects [133]. At present, the available evidence is limited to preclinical studies, which suggest that its protective effects may vary depending on the type of extract used, the dosage, and the duration of treatment.

In experimental rat models, short-term administration of ethanolic extracts at a dose of 200 mg/kg for 7–10 days was found to reduce chemically induced hepatic inflammation and congestion. These findings indicate that *C. colocynthis* may exhibit hepatoprotective activity under controlled experimental conditions [133,134]. Similarly, *C. colocynthis* extracts attenuated carboplatin- and thalidomide-induced hepatotoxicity by enhancing antioxidant defenses (GSH and NRF2) and restoring mitochondrial biogenesis regulators (PGC-1α and mtTFA) [135].

Despite these promising findings, the available evidence has several important limitations. To date, all studies have been conducted in animal models, and there is considerable variation in extract preparation, treatment protocols, and experimental designs, making it difficult to directly compare the findings across studies. In addition, long-term administration studies have shown that although doses of 50–100 mg/kg did not cause obvious signs of chronic toxicity, treatment for three months resulted in an increase in liver weight, suggesting the possibility of cumulative effects on the liver [136].

Overall, the current evidence suggests that *C. colocynthis* may have hepatoprotective effects under certain experimental conditions. However, its narrow therapeutic window and documented hepatotoxicity indicate that its use should be approached with caution. Further research, including comprehensive dose–response studies, standardized characterization of extracts, long-term toxicological assessments, and clinical investigations, is needed before its hepatoprotective potential can be established with greater confidence.

### 5.12. Cardioprotective Activity

Evidence supporting the cardioprotective activity of *C. colocynthis* is currently limited to a small number of preclinical animal studies and should therefore be interpreted with caution [137,138,139]. The reported effects vary according to the extract type, experimental model, and measured cardiovascular endpoints.

In an adrenaline-induced myocardial injury rabbit model, a hydroalcoholic peel extract reduced cardiac tissue damage compared with untreated animals [138]. Similarly, in a doxorubicin-induced cardiotoxicity rat model, a hydro-alcoholic leaf extract dose-dependently reduced serum cardiac injury biomarkers, including creatine kinase-MB (CK-MB), lactate dehydrogenase (LDH), and cardiac troponin I, while preserving myocardial histoarchitecture. These protective effects have been attributed primarily to antioxidant and anti-inflammatory mechanisms that reduce oxidative stress and stabilize myocardial cell membranes [139].

In another study, an ethyl acetate fraction enriched with polyphenolic compounds, including myricetin and quercetin derivatives, significantly reduced blood pressure and improved electrocardiographic parameters in hypertensive rats. Although this fraction also demonstrated strong antioxidant activity in the DPPH assay, these in-vitro findings should not be interpreted as the direct mechanism underlying the observed cardiovascular effects in-vivo [137].

Overall, the available evidence suggests that *C. colocynthis* possesses cardioprotective and antihypertensive potential in experimental animal models. However, the current evidence remains limited by the absence of clinical studies, variability in extract composition, unknown human bioavailability, and insufficient long-term safety evaluation [140,141]. Therefore, standardized extracts, mechanistic investigations, comprehensive toxicological studies, and well-designed clinical trials are required before its cardioprotective potential can be reliably established.

## 6. Application of *C. colocynthis* in Poultry

Preliminary studies have investigated the use of different parts, powders, and extracts of *C. colocynthis* as dietary supplements in poultry. These studies suggest that seeds and fruits contain bioactive constituents, including polyphenols, flavonoids, and polyunsaturated fatty acids (predominantly linoleic acid), which may contribute to antioxidant and immunomodulatory effects [1]. Experimental studies have reported improvements in productive performance and reductions in oxidative stress markers in laying hens following dietary supplementation with seed extracts or powders. Similarly, fruit powder and fruit pulp have been associated with improved body weight gain and carcass yield in broiler chickens (Table 6) [3,26,142,143,144,145]. Several studies from Kuwait have proved the beneficial use of plant materials [146,147] and phytogenic feed additives [148] when used to feed the poultry in harsh environmental conditions. Similar to these plants, *C. colocynthis* has a strong potential to be used as a locally available poultry feed resource under arid climatic conditions. Also, early nutritional management has been shown to lessen heat-stress-related physiological disruption in broilers and improve production stability [149]. Antioxidant supplementation combined with feed-management strategies can further improve thermotolerance under heat stress [150].

The agricultural potential of *C. colocynthis* should therefore be evaluated alongside its well-documented toxicological profile. Although several studies have reported beneficial effects under controlled experimental conditions, these findings are insufficient to support its routine application as a commercial poultry feed additive [151]. Safe dietary inclusion levels remain inadequately defined, and excessive intake of bioactive constituents, particularly cucurbitacins, may result in hepatotoxicity, nephrotoxicity, gastrointestinal disturbances, and reduced animal performance [152]. Consequently, further studies are required to establish standardized processing methods, determine safe dietary inclusion levels, and evaluate long-term safety before practical application in poultry production.

Overall, *C. colocynthis* represents a promising candidate for functional poultry nutrition; however, its practical application requires comprehensive toxicological evaluation, dose standardization, and long-term in-vivo studies to ensure animal safety and prevent the transfer of potentially harmful metabolites into the human food chain.

### Anti-Nutritional Factors and Detoxification Strategies

Despite the reported improvements in broiler and layer performance, the use of *C. colocynthis* in poultry diets requires careful consideration because of its anti-nutritional factors (ANFs). The seeds and fruit pulp contain cucurbitacins, saponins, tannins, and other bioactive compounds that may interfere with nutrient utilization when present at high concentrations [7,30,153]. These compounds can affect digestive enzyme activity and reduce nutrient digestibility, which may negatively influence growth performance and overall animal health [30,154].

To reduce these potential adverse effects, raw *C. colocynthis* biomass should be appropriately detoxified before being incorporated into poultry diets. Processing techniques such as roasting, boiling, autoclaving, prolonged soaking in water, and solvent extraction have been reported to reduce the levels of toxic constituents and improve feed safety [7,30]. However, experimental evidence on the most effective detoxification methods and safe dietary inclusion levels is still limited. Available studies generally recommend relatively low inclusion rates (approximately 0.1–2.0%, depending on the plant part and preparation used) [3,26,143]. Exceeding these levels, particularly when unprocessed or inadequately detoxified materials are used, may lead to gastrointestinal disturbances, impaired growth performance, and toxic effects on internal organs [153,154]. Therefore, further studies are needed to standardize detoxification protocols, establish evidence-based inclusion levels, and evaluate long-term safety under commercial poultry production conditions.

## 7. Toxicity Profile and Safety Considerations

Although *C. colocynthis* exhibits diverse biological activities and has a long history of traditional medicinal use, its clinical and agricultural applications are severely limited by its narrow therapeutic index (NTI) and well-documented toxicity [155]. Assessment of its safety requires integration of clinical intoxication reports with findings from experimental toxicological studies. Adverse effects are most commonly associated with the consumption of raw fruit pulp, concentrated extracts, or excessive quantities of plant material used in traditional medicinal preparations [155,156].

The most commonly reported adverse effects in humans involve severe gastrointestinal toxicity. In one documented clinical case, a 37-year-old man developed severe vomiting, abdominal pain, abdominal tenderness, and hematochezia after consuming *C. colocynthis* fruit extract as a digestive remedy [47]. These symptoms are consistent with the strong gastrointestinal irritant effects associated with the plant.

Experimental toxicological studies have shown that the toxicity of *C. colocynthis* can vary considerably depending on the plant part used, extraction method, phytochemical fraction, dose, and duration of exposure. Reported LD_50_ values also differ substantially among different preparations. For instance, isolated saponins showed an LD_50_ of 200 mg/kg in mice [153], whereas crude extracts had an LD_50_ of approximately 3903.2 mg/kg [157]. These findings suggest that purified phytochemical fractions, particularly saponins and cucurbitacins, may be considerably more toxic than crude aqueous or ethanolic extracts.

At higher doses, *C. colocynthis* has consistently been associated with entero-hepato-nephrotoxicity in experimental animals [154]. Reported toxic effects include significant increases in hepatic enzyme levels, degeneration of renal tissue, fatty changes in epicardial tissue, and extensive histopathological damage to the liver, kidneys, and intestines [28,154]. The toxic effects may be further intensified when *C. colocynthis* is administered in combination with other toxic plants. For example, co-administration with *Nerium oleander* resulted in a synergistic increase in toxicity and mortality in experimental rats [158].

Overall, the available evidence does not support the unrestricted use of *C. colocynthis* as a medicinal preparation or livestock feed. Although the plant has demonstrated promising pharmacological properties, its narrow therapeutic window and potential for both acute and chronic toxicity warrant considerable caution. Future applications will require standardized extract preparation, comprehensive dose–response characterization, long-term toxicological evaluation, and rigorous safety assessment before clinical or agricultural use can be considered.

## 8. Rangeland Development

*C. colocynthis* is well adapted to arid and semi-arid environments and can tolerate prolonged drought, high temperatures, and the low fertility of sandy soils [159,160]. These adaptations are supported by its deep taproot, which enables access to subsurface water, and its prostrate growth habit, which minimizes transpirational water loss. While these traits contribute to the species’ survival under harsh environmental conditions, they should not be directly interpreted as evidence of large-scale rangeland restoration potential.

The extensive root system of *C. colocynthis* may contribute to soil stabilization and help reduce wind and water erosion in sandy habitats [160,161]. However, quantitative field evidence demonstrating its effectiveness in large-scale rangeland rehabilitation remains limited. Likewise, its influence on soil water dynamics, soil fertility, plant community succession, and interactions with co-occurring vegetation has not been adequately investigated. In particular, whether *C. colocynthis* functions as a nurse species facilitating native plant establishment or competes with more palatable forage species under resource-limited conditions remains unclear.

Therefore, the current evidence is insufficient to recommend *C. colocynthis* for large-scale rangeland development or restoration programs. Although its xerophytic adaptations and ability to colonize degraded habitats suggest potential ecological value, its role in ecosystem restoration remains largely speculative. Future research should focus on long-term field evaluations, community-level ecological interactions, soil health, and ecosystem functioning before the species can be reliably incorporated into sustainable rangeland rehabilitation strategies.

### 8.1. Nabkha Formation

Among many plants in desert area, *C. colocynthis* stands out as one of the plant capable to grow desert nabkhas and trap mobile sediment [160]. The plant is seen to effectively trap the sand when it grows at least 15 cm, and show an efficacy to trap 1 to 2 m^3^ sand and generate larger and longer nabkhas [159]. These nabkhas act as natural windbreaks and reduce wind erosion and stabilize loose sandy soil, which is major factor in rangeland development. The seeds disperse naturally and germinate in the same location, contributing to the sustainable formation of nabkhas. The fruit generates many seeds, and this plant proves to be better suited for rangeland development [159,160]. Unfortunately, this plant has never been studied to be a potential candidate for rangeland development.

### 8.2. Water Retention and Drought Resilience

This plant makes up a good storage of water in its fruit. This helps to withstand prolonged drought conditions. The ability of *C. colocynthis* to survive prolonged drought conditions is partly attributed to its deep root system, which allows access to subsurface moisture [42]. This not only allows plant survival, but also helps to maintain soil moisture levels, supporting other plants to grow alongside and establish green spots in rangelands. This in turn happens to be one of the factors for development of nabkhas. Limited data exist for its impact on soil moisture retention and facilitation of other plant species. The vegetation of *C. colocynthis* forms an average land cover with the leaves, which reduces the soil temperature below the plant. In places like Kuwait, where water availability is low, the presence of *C. colocynthis* can contribute significantly to improving water retention in the soil and reducing surface evaporation [160].

### 8.3. Seed Dispersal and Artificial Rangeland Development

*C. colocynthis* produces a high number of seeds, making it well-suited for large-scale rangeland restoration programs. While the seeds naturally germinate in their designated season, large-scale germination efforts require scarification treatment to enhance seed viability and promote uniform growth [160]. Complementary soil-amendment strategies such as biochar are increasingly discussed as tools for sustainable poultry-linked resource cycles and dryland restoration [161]. Seed scarification treatments, including mechanical abrasion, acid treatment, or hot water immersion, have been shown to break dormancy and increase germination rates. In Kuwait, and the neighboring countries targeted seed dispersal programs and controlled germination techniques can be employed to propagate this species effectively across degraded rangelands. Despite bitter taste, many grazing animals accidentally eat the fruits or seeds, which naturally pass through the digestive tract and often pass unharmed though stools. This phenomenon also helps in seed dispersal, where it provides a probability of new nabkhas formation. As per the available report, *C. colocynthis* fruit at 1% concentration in the fodder showed potential of improving rumen fermentation and reducing methanogenesis [27]. This makes *C. colocynthis* candidate plant for therapeutic and fodder purpose. The seeds of this plant are proven to be immunomodulatory for increasing the performance of broiler chickens under heat stress conditions [143]. These benefits highlight the increasing need to develop rangelands for cultivating this plant and harvesting its seeds as animal feed or supplements.

## 9. Challenges and Future Perspective

Although *C. colocynthis* has long been used in traditional medicine and demonstrates diverse pharmacological activities in experimental studies, its translation into evidence-based therapeutic and agricultural applications remains limited by several unresolved challenges. Current evidence is characterized by considerable heterogeneity in plant materials, extraction procedures, phytochemical composition, experimental models, and safety assessments, making direct comparison among studies difficult. Future research should therefore move beyond descriptive studies and adopt standardized, multidisciplinary approaches that bring together phytochemistry, pharmacology, toxicology, and clinical research.

### 9.1. Phytochemical Standardization and Quality Control

One of the major challenges in the therapeutic development of *C. colocynthis* is the considerable natural variation in its phytochemical composition, particularly in the levels of cucurbitacins and other bioactive metabolites. These variations can be influenced by geographical origin, environmental conditions, cultivation practices, harvesting stage, and extraction methods [24]. Differences in phytochemical composition may consequently lead to inconsistent pharmacological effects and unpredictable toxicity, making it difficult to reproduce findings across studies [162]. Future research should therefore focus on standardized cultivation practices, proper authentication of plant materials, validated extraction procedures, and comprehensive phytochemical profiling before biological evaluation. Modern extraction techniques, such as ultrasound-assisted extraction, microwave-assisted extraction, and supercritical fluid extraction, may help produce more consistent extracts with improved reproducibility while reducing the presence of undesirable toxic constituents.

### 9.2. Rigorous Toxicological Profiling and One Health Safety Assessment

Despite its promising biological activities, the narrow therapeutic index of *C. colocynthis* remains a major concern for both medicinal and nutritional applications. Comprehensive toxicological studies are needed to establish safe dosage ranges, identify target organs affected by toxicity, assess the effects of long-term exposure, and determine the potential for herb–drug interactions. From a One Health perspective, safety assessments should also consider the possibility of toxic metabolites being transferred to animal-derived food products when *C. colocynthis* is used as livestock or poultry feed [16,146,147,148,163,164]. Future research should therefore integrate toxicology, veterinary science, environmental safety, and food safety to establish evidence-based safe inclusion levels for both pharmaceutical and agricultural applications.

### 9.3. Mechanistic Validation and Clinical Translation

Although numerous in vitro and animal studies have suggested potential mechanisms underlying the pharmacological activities of *C. colocynthis*, clinical evidence remains limited. Most available human studies have involved small sample sizes, short intervention periods, and different types of plant preparations, making it difficult to draw firm conclusions about its efficacy and safety. Future research should place greater emphasis on mechanistic studies aimed at identifying and validating specific molecular targets, along with well-designed randomized controlled clinical trials using standardized preparations and clearly defined dosing regimens. Such studies are essential to determine whether the observed preclinical benefits can be translated into safe and effective clinical applications [7,8].

### 9.4. Sustainable Cultivation and Ecological Assessment

The high drought tolerance of *C. colocynthis* makes it a promising candidate for cultivation in arid and semi-arid regions, particularly in areas such as Kuwait and other Middle Eastern countries where water availability is limited [23,41]. However, sustainable large-scale cultivation should be evaluated not only in terms of agronomic performance but also from an environmental perspective. Future studies should examine water-use efficiency, long-term effects on soil health, ecological interactions, impacts on biodiversity, and cultivation practices that minimize environmental disturbance. Developing sustainable production systems supported by efficient irrigation technologies and environmentally responsible agricultural practices will be important for ensuring a reliable and ecologically sustainable supply of standardized plant material [165].

## 10. Conclusions

*C. colocynthis* is a phytochemically rich medicinal plant containing a wide range of bioactive compounds with promising pharmacological and nutritional potential, as demonstrated mainly through in vitro and preclinical studies. However, the available evidence is still limited by differences in research methods, a lack of phytochemical standardization, limited clinical validation, and concerns about dose-dependent toxicity and its narrow therapeutic index. The current evidence is not yet sufficient to support its routine therapeutic or agricultural use. Future research should focus on standardized extract preparation, thorough toxicological evaluation, mechanistic studies, and well-designed clinical and translational research to establish its safety and effectiveness. With proper scientific validation, *C. colocynthis* may have valuable applications in pharmaceutical, veterinary, and agricultural fields.

## Figures and Tables

**Table 1:** A detailed list of the chemical compounds in *C. colocynthis*.

Name of Compounds	Part of Plant	Solvent for Extraction	Method for Isolation and Identification	Reference
**Curcubitacin and its glycosides**				
Curcubitacin B, & E	Leaves	70% Chloroform/Methanol	Thin-Layer Chromatography (TLC) and NMR	[64]
Curcubitacin E	Fruits	*n*-butanol	HPLC	[64]
Curcubitacin E, I, J, K, L, Colocynthosides A & B	Fruits	Methanol	RP-Silica Gel Column Chromatograph, HPLC, FAB-MS	[65]
Norcolocynthenin A & B	Fruits	Methanol	NMR	[66]
2-*O*-*β*-*D*-glucopyranosyl-16-20R-dihydroxy-cucurbita1,5,23E,25(26)-tetraen-3,11,22-trione	Fruits	Methanol	TLC & NMR	[67,68]
2-*O*-*β*-*D*-glucopyranosyl-cucurbitacin B (arvenin I)				
2,25-di-O-β-D-glucopyranosyl-cucurbitacin L				
2-*O*-*β*-*D*-glucopyranosyl cucurbitacins K, J, & I	Fruits	70% Ethanol	NMR	[69]
6′-acetyl-2-O-β-D-glucocucurbitacin E	Leaves	Ethyl acetate	NMR and MS	[70]
25-*p*-coumaroyl-3′-acetyl-2-*O*-*β*-*D*-glucocucurbitacin I				
**Alkaloids**				
Quinolone, 2-hydroxyquinoline, 4-hydroxyquinoline, 6-hydroxyquinoline, 8-hydroxyquinoline, 2-methylquinoline, 4-methylquinoline, 6-methylquinoline, 8-methylquinoline, & 7,8-benzoquinoline	Fruits	Methanol	HPLC	[71]
**Flavonoids**				
Isosaponarin, isovitexin, isoorientin 3′-*O*-methyl ether	Fruits	Methanol	HPLC	[72]
Quercetin, Myricetin, Kaempferol, Catechin	Roots, leaves, and fruits	*n*-hexane and ethanol	HPLC	[73]
**Steroids**				
Spinasterol, & 22,23-dihydrospinasterol	Leaves	-	NMR and MS	[74]
**Phenolic acids**				
Ferulic acid, *p*-hydroxybenzoic acid, hydroxycaffeic acid, caffeic acid, *p*-coumaric acid	Fruit (coat, flesh) & seed	Methanol	HPLC	[44]
4-dihydroxy-phenyl-acetic acid, syringic acid, phenylalanine	Fruit coat and seed			
Hydroxy-4-methylcoumarin	Fruit coat			
Protocatechuric acid	Seeds			
Gallic acid, chlorogenic acid, ferulic acid, vanillic acid, *p*-coumeric acid, sinapic acid, *p*-hydroxy benzoic acid, caffeic acid	Roots, leaves, and fruits	*n*-hexane and ethanol	HPLC	[73]
**Fatty acids**				
Linoleic acid, oleic acid, palmitic acid & stearic acids	Seeds	Oil	NMR and FTIR	[75]
**Tocopherols**				
*α*-tocopherol, *β*-tocopherol, *γ*-tocopherol, & *δ*-tocopherol	Seeds	Oil	NMR and FTIR	[75]

**Table 2:** In-vitro anti-cancer activity of various extracts and phyto-constituents of *C. colocynthis*.

Extract/Phytocompound	Cell Line	Result	Ref.
Methanolic extract of leaves	MCF-7	LC_50_ = 30 ± 4.15 μg	[78]
Ethanolic extract of Fruits	MCF-7, MDA-MB-231, SiHa cell line	LC_50_ = 105 ± 8.3, 142 ± 16, & 157 ± 4.3 µg/mL	[79]
Acetone extract of Fruits	MCF-7, MDA-MB-231, SiHa cell line	LC_50_ = 94 ± 6.4, 140 ± 0.8 & 121 ± 4.6 µg/mL	
Methanolic extract of leaves	MCF-7	LC_50_ = 30 μg	[80]
Seed oil	Caco-2, HCT−116, Normal skin fibroblast	IC_50_ = 7 ± 0.9, 4 ± 0.3, 88 ± 6 mg/mL	[81]
Cucurbitacin E	Drug sensitive strains: CCRF-CEM, HCT116 p53^+/+^, U87.MG, HEK293 & MDA-MB-231	IC_50_ = 0.32 ± 0.04, 8.29 ± 2.29, 73.4 ± 9.8, 17.02 ± 4.1, 0.009 ± 0.007 nM	[82]
	Drug resistance strains: CEM/ADR5000, HCT116 p53^−/−^, U87.MGΔEGFR, HEK293 ABCB5, MDA-MB-231 BCRP	IC_50_ = 1.3 ± 0.5, 13.72 ± 2.02, 1.3 ± 0.4, 7.9 ± 6.9, 0.09 ± 0.9 nM	
Cucurbitacin B glucoside	ER+ MCF-7 human breast cancer cell	Treated cells showed rapid reduction in the level of key protein complex necessary for regulation of G2 exit and initiation of mitosis, namely the p34CDC2/cyclin B1 complex.	[64]
	ER MDA-MB-231 human breast cancer cell		
Cucurbitacin E glucoside	HepG2 hepatoma cells	IC_50_ = 3.5 µM	[83]
Cucurbitacin I glucoside		IC_50_ = 2.8 µM	
25-*p*-coumaroyl-3-acetyl-2-O-β-D glucocucurbitacin I	Human colon cancer cell lines (Caco-2)	−19%	[70]
	Human colon cancer cell lines (HT29)	−32%	
Norcolocynthenins A & B	HL-60	IC_50_ = 8.32 & 6.49 µM	[67]

**Table 3:** In-vitro broad-spectrum biocidal and antiparasitic activity of various extracts and phyto-constituents of *C. colocynthis*.

Extract/Phytocompound	Strain	Result	Ref.
Methanolic extract of fruits	CQ-sensitive and resistant strains (3D7 and K1, respectively)	IC_50_ = 2.01 and 6.9 µg/mL, respectively	[87]
Methanolic extract of fruits	*Toxoplasma gondii* RH-GFP *&* human foreskin fibroblast (HFF) cells	IC_50_ = 22.86 & 27.74 µg/mL	[88]
Petroleum ether extract of leaves	*Aedes aegypti* & *Culex quinquefasciatus*	LC_50_ = 74.57 & 88.24 ppm LC_90_ = 538.30 & 337.90 ppm	[89]
Ethanolic extract of leaves	*Brevicoryne brassicae* L.	LC_50_ = 6.26 mg/mL via residual & 0.22 mg/mL via contact assay	[90]
Benzene, petroleum ether, ethyl acetate, & methanolic extract of leaves	*C. quinquefasciatus* (*Say*)	LC_50_ = 61.72, 66.92, 47.58, & 118.74 ppm LC_90_ = 109.98, 131.37, 88.79, & 243.02 ppm	[91]
Ethyl acetate extract of leaves	*Mollusc gastropod*	LC_50_ = 12.6 mg/mL	[92]
Cucurbitacin E	*Galba truncatula*	LC_50_ = 9.55 µg/mL	[92]
Cucurbitacine 2-o-β-d-glucopyranoside		LC_50_ = 10.61 µg/mL	
Linoleic acid	*A. aegypti* L., *Anopheles stephensi Liston*, & *C. quinquefasciatus Say*	LC_50_ = 18.20, 11.49, & 27.24 ppm LC_90_ = 96.33, 47.35, & 70.38 ppm	[93]
Oleic acid	*A. aegypti* L., *A. stephensi Liston*, & *C. quinquefasciatus Say*	LC_50_ = 8.80, 9.79, 7.66 ppm LC_90_ = 35.39, 37.42, 30.71 ppm	
Spinasterol	*Brevicoryne Brassicae* L.	LC_50_ = 37.50 µg/mL	[94]
7,8-benzoquinoline	*Tetranychus urticae*, *Sitophilus oryzae*, *Sitophilus zeamais*	LC_50_ = 11.8, 2.9, 2.7 µg/mL	[71]

**Table 4:** In-vitro anti-microbial activity of various extracts and phyto-constituents of *C. colocynthis*.

Extract/Phytocompound	Strain	Result	Ref.
Ethyl acetate extract of leaves	*Staphylococcus. aureus*	MIC = 0.625 mg/mL	[95]
Gluco-cucurbitacin E	*Bacillus cereus* & *Enterococcus faecalis*	MIC = 1.25 mg/mL	
Ethanolic extract of seed, root & fruits	*S. aureus*, *Bacillus subtilis*, *Pseudomonas aeruginosa*, *Klebsiella pneumoniae*, *Candida albicans*, *Aspergillus fumigatus*, *Aspergillus niger*, *Fusarium oxysporum*	ZOI = 9–22, 7–23 & 9–23 mm	[96]
Methanolic extract of seeds	*S. aureus*	MIC = 5 mg/mL and MBC =10 mg/mL	[97]
Aqueous extract of leaves	*B. subtilis*, *S. aureus*, *Streptococcus pyogenes*, *Escherichia coli*, *K. pneumoniae*, *Salmonella typhi*, *Mucor* sp., *Penicillium* sp.	ZOI = 2–14 mm	[98]
Methanolic extract of leaves	*B. subtilis*, *S. aureus*, *Streptococcus faecalis*, *Streptococcus pyogenes*, *E. coli*, *Proteus mirabilis*, *S. typhi*, *A. fumigatus*, *Mucor* sp.	ZOI = 1–13 mm	
Methanolic extract of ripe deseeded fruit	*Mycobacterium tuberculosis* H37Rv (ATCC 27294), 16 clinical isolates of *M. tuberculosis* (MDAM 009-001 to 16), 2 clinical isolates of *MOTT* (MDAM 009-0025 to 26)	MIC = ≤62.5 µg/mL	[99]
Aqueous extract of fruit	*E. coli*, *S. aureus*, *S. typhi*, *S. shigella*, *C. albicans*	ZOI = 1–7 mm	[100]
Methanolic extract of fruit	*S. aureus*, *S. typhi*, *S. shigella*, *C. albicans*	ZOI = 1–8 mm	
Petroleum ether extract of fruit	*C. albicans*	ZOI = 1 mm	
Ethanolic extract of fruit	*E. coli*, *S. aureus*, *S. typhi*, *S. shigella*, *C. albicans*	ZOI = 4–9 mm	
Acetone extract of fruit	*E. coli*, *S. aureus*, *S. typhi*, *S. shigella*, *C. albicans*	ZOI = 2–16 mm	
Ethanolic and aqueous extract of fruit or leaves	*S. aureus* standard strain (ATCC 25923) and *S. aureus* isolate from patients	ZOI = 20 mm & 2–12 mm	[101]
Aqueous extract of fruit	*S. aureus*, *Streptococcus* sp., *B. subtilis*, *E. coli*, *Klebsiella* sp.	MIC = 600, 700, 1500, 3000, 1000 µg/mL	[102]

MIC, minimum inhibitory concentration; ZOI, zone of inhibition; MBC, minimum bactericidal concentration.

**Table 5:** Anti-diabetic activity of various extracts of *C. colocynthis*.

In-Vitro Studies							
Extract/Phytocompound		Assay	Concentration		Result		Ref.
Fruit extract		3T3-L1 adipocytes	4, 20, or 100 μg/mL		Insulin-enhancing activity		[108]
Fruit extract		Human hemoglobin	0, 0.1, 0.3, 0.5, 1 g/dL		Significant decrease in the formation of HbA1C		[109]
Aqueous extract of seed		Rat pancreatic islets	45, 135, 450 & 4500 µg/mL		45 µg/mL extract significantly increased insulin output at all D-glucose concentrations (2.8, 8.3 and 16.7 mM).		[110]
Hydro-ethanolic extract of pulpy flesh with seeds		α-glucosidase	0.5, 0.12 & 0.25 mg/mL		Extract inhibited α-glucosidase that is responsible for postprandial hyperglycemia.		[111]
Crude defatted, and purified extract of seeds		Isolated rat pancreatic islets	0.1 mg/mL		Extract Stimulates Insulin Secretion		[112]
**Animal experimentation**							
**Extract**	**Animals**	**Dose**	**Duration**	**Disease Inducer**	**Standard Drug**	**Result**	**Ref.**
Aqueous extract of fruit	Rabbits	300 mg/kg	-	Alloxan (150 mg/kg i.v.)	-	Reduction in plasma glucose after 1 h & highly significant after 2,3 and 6 h	[55]
Alkaloids, glycosidic, and saponin extract		10, 15 and 20 mg/kg				Glycosidic and saponin extract demonstrated significant reduction in plasma glucose after 1 h and highly significant after 2, 3 and 6 h	
Crude or defatted aqueous extract	Male wistar rats	90 mg/kg	Short term experiment = 0 to 180 min Long term experiment = 3 weeks	Streptozotocin (65 mg/kg, i.p.)	Glibenclamide (10 mg/kg, i.p.)	Lowered glycemia, serum triglyceride level, prevent progressive increase in serum cholesterol level, epididymal fat weight and lesser decrease in body weight	[113]
H_2_O-methanol extract		100 mg/kg					
Ethyl acetate extract		10 mg/kg					
*n*-butanol extract		70 mg/kg					
Aqueous extract of seed	Female wistar rats injected	90 mg/kg	21 days	Streptozotocin (0.25 mmol/kg, i.p.)	-	Extract minimized the severity of hyperglycemia	[114]
Hydro-ethanolic extract of pulpy flesh with seeds	Male wistar albino rats	150 and 300 mg/kg	14 days	Streptozotocin (55 mg/kg, i.p.)	Glibenclamide (600 µg/kg)	Extract reduced the blood glucose, cholesterol and triglyceride levels.	[111]
**Clinical Trials**							
**Treatment**	**Type of Trial**	**Dose**	**No. of Patients**	**Groups**	**Treatment Duration**	**Result**	**Ref.**
*C. colocynthis* powder	Single Arm	decoction containing 2% CCT solution	40 patients with type II diabetes	-	10 days	reduction in fasting blood glucose levels, stimulation of serum insulin secretion, improvement of pancreatic beta-cell function and reduction in serum urea levels.	[115]
*C. colocynthis* fruit	double-blind clinical trial	125 mg whole dried fruit powder per capsule	70 patients with type II diabetes	Test and Placebo (35 each)	2 months	Significantly decrease in mean serum levels of HbA1c and FBS among patients with type II diabetes.	[116]
*C. colocynthis* fruit	randomized, double blind, placebo-controlled clinical trial	100 mg *C. colocynthis* fruit capsules three times a day	50 patients with type II diabetes	Test and Placebo (25 each)	2 months	Significant decrease in HbA1c and fasting blood glucose levels in *C. colocynthis* treated patients	[117]
*Gymnema sylvestre*, *C. colocynthis*, and *Artemisia absinthium*	-	2 capsules (500 mg)	30 patients with type II diabetes	*G. sylvestre*, *C. colocynthis*, & *A. absinthium* (8 each)	40 days	*G. sylvestre* reduced 37% glucose, 5% TGL, 13% cholesterol and 19% LDL level. *C. colocynthis* reduced glucose, cholesterol and TGL and LDL-cholesterol level by 35, 6, 6, and 5%. *A. absinthium* reduced 3% HDL and 6% LDL level.	[118]

**Table 6:** Use of *C. colocynthis* in poultry.

Plant Part/Form	Dose/Inclusion	Poultry Type	Main Effects	Ref.
Hydroethanolic extract of seed	1g/kg feed	Hy-line brown laying hens	Enhance layers production and physiological performances.	[142]
Ethanolic extract of seed	0.5, 1.0, and 2.0 g/kg	Hy-line brown laying hens	Improved the productive and physiological performance	[143]
Fruit powder	1.0 & 1.5 g/kg feed	Ross-308 broiler chicks	Weight gain	[3]
Fruit pulp	0.75, 1.5, 2.25 and 3 g/kg	Ross-308 old broiler chicks	Improves growth	[26]
Seed powder	1% diet (10 g/kg)	Hy-line brown layers (oxidative stress)	Increase in egg number and egg mass, reduces stress biomarkers	[144]
Seed	2, 4, & 6% diet	Broiler chickens	Improves body weight, carcass yield	[145]

## Data Availability

No new data were created or analyzed in this study. Data sharing is not applicable to this article.

## Abbreviations

ABTS	2,2′-azino-bis(3-ethylbenzothiazoline-6-sulfonic acid)
ALT	Alanine Aminotransferase
ANFs	Anti-Nutritional Factors
AST	Aspartate Aminotransferase
BHT	Butylated hydroxytoluene
CK-MB	Creatine Kinase-MB (CK-MB)
CVDs	Cardiovascular Diseases
DPPH	2,2-diphenyl-1-picrylhydrazyl
EAF	Ethyl Acetate Fraction
FBG	Fasting Blood Glucose
FTIR	Fourier-transform infrared spectroscopy
HbA1c	Glycated Hemoglobin
HPLC	High-Performance Liquid Chromatography
ICBA	International Center of Biosaline Agriculture
LDH	Lactate Dehydrogenase
LD_50_	Median Lethal Dose
MDA	Malondialdehyde
MIC	Minimum Inhibitory Concentration
MS	Mass Spectrometry
NTI	Narrow Therapeutic Index
NMR	Nuclear Magnetic Resonance
mtTFA	Mitochondrial Transcription Factor A
NRF2	Nuclear Factor Erythroid-2-Related Factor 2
NSAIDs	Non-Steroidal Anti-Inflammatory Drugs
PCA	Passive Cutaneous Anaphylaxis
PCD	Preconvulsion Dyspnea Time
PGC-1α	Proliferator-Activated Receptor-Gamma Coactivator-1 Alpha
SOD	Superoxide Dismutase
TLC	Thin-Layer Chromatography.
ZOI	Zone of Inhibition
